# Self-assembled Cube-like Copper Oxide Derived from a Metal-Organic Framework as a High-Performance Electrochemical Supercapacitive Electrode Material

**DOI:** 10.1038/s41598-019-45557-6

**Published:** 2019-06-24

**Authors:** Abdullah Aljaafari, Nazish Parveen, Faheem Ahmad, Mir Waqas Alam, Sajid Ali Ansari

**Affiliations:** 1 Department of Physics, College of Science, King Faisal University, AlAhsa, 31982 Saudi Arabia; 2 Deparment of Chemistry, College of Science, King Faisal University, AlAhsa, 31982 Saudi Arabia

**Keywords:** Electrocatalysis, Chemistry, Condensed-matter physics, Nanoscience and technology

## Abstract

Interest in pseudocapacitive materials, especially cuprous oxide, has grown owing to its various advantageous properties and application as electrode materials in the energy storage devices. The work presented here, a cubic Cu_2_O framework was synthesized using a simple and one-step modified polyol-assisted (metal-organic framework) solvothermal method. The structural configuration was rationalized by systematically studying the effect of the reaction time on the morphology and growth of the Cu_2_O. In addition, a range of microscopic and spectroscopic techniques was employed to further characterize the obtained cubic Cu_2_O. The morphological effect on the electrochemical supercapacitive performance of the obtained cubic Cu_2_O was also examined by cyclic-voltammetry (CV) and galvanostatic-charge-discharge (G-C-D) method. The obtained outcome shows that the cubic Cu_2_O synthesized using a reaction time of 12 h (Cu_2_O-12h; C_sp_ ~365 Fg^−1^) exhibited superior capacitive performance as compared to the cubic Cu_2_O synthesized at 8 h (Cu_2_O-8h; C_sp_ ~151 Fg^−1^) and 10 h (Cu_2_O-10h; C_sp_ ~195 Fg^−1^) at the current density of 0.75 Ag^−1^. Furthermore, the Cu_2_O-12h electrode exhibits energy density of 16.95 Wh/Kg at a power density of 235.4 W/Kg and higher power density of 2678.5 W/Kg at low current density. In particular, the cube-like Cu_2_O-12h exhibited excellent capacitive performance and rate capability as compared to Cu_2_O-8h and Cu_2_O-10h, owing to its unique three-dimensional morphology, which facilitates the formation of various active sites for intercalation of the electrolyte during the electrochemical process. These results show the as-obtained Cu_2_O could be a promising supercapacaitive electrode material for various applications.

## Introduction

The devastating incensement of the human population and industrial development over the past years has caused in the extreme exploitation of naturally produced fossil fuels to fulfill the upward energy requirements. The resultant excessive use of fossil fuels causes pollution and the depletion of these fuels. To overcome this problem, various research has been focused to develop a renewable sources of clean energy and the enhancement of energy conversion and energy storage. In many application areas such as supercapacitors (electrical double-layer capacitors and pseudocapacitors), batteries (sodium-ion and lithium-ion batteries) and fuel cells serve as the most practical and active technologies for energy conversion and energy storage^1–8^. Among these technologies, electrochemical supercapacitor or supercapacitor have got lot of attention due to their fast charging-discharging capability and high power density, and long life span, which can be used as perfect intermediate between the traditional electrochemical capacitor and batteries^9^. Based on the energy storage mechanism, electrochemical-supercapacitor can be classified as electric-double-layer-capacitor (EDLCs) which includes carbon based materials such as carbon nanotubes, graphene and graphene oxide, etc. These EDLCs release and stores energy through accumulating charged particles at the surface of the electrode and electrolyte interfaces. This mechanism behaves as a pseudocapacitor, which contains conducting polymers and metal oxides, and which stores energy in the form of a faradic redox reaction with the electrode materials. Among these electrode materials, metal oxides (Co_3_O_4_, MnO, CuO, NiO, Fe_2_O_3_) have been widely used in research because of their benefits such as their low cost, effective faradic reaction, and high availability^10–12^. However, most of these metal oxides exhibit poor electronic conductivity and have a short lifespan, which restricted their use in high energy density based supercapacitor. To overcome these issues various carbon based materials which comprises high surface area and high electronic conductivity, are combined with metal oxides. Furthermore, the use of battery type materials with faradic current properties profile another approach to fabricate a high performance supercapacitor. In this regard, among the aforementioned transition-metal oxide, copper oxide is of interest due to its interesting physicochemical properties, affordability, environmentally friendly nature, and its nontoxicity. In particular, cupric (CuO) and cuprous oxide (Cu_2_O) are effective due to the semiconductor behavior and are advantageously used in certain application such as photocatalysis, solar cell, supercapacitor applications, and lithium-ion batteries^13–15^. The application of Cu_2_O in energy storage device suggest the feasibility of its in supercapacitor application due to its similar faradic behavior known to occur in both lithium ion batteries and pseudocapacitors^16^. Cupric oxide has been widely used in supercapacitor applications^17–19^. Dubal *et al*. synthesized cauliflower-shaped CuO particles for supercapacitor application with specific capacitance of 179.0 Fg^−1^ in 1 M Na_2_SO_4_ electrolytic solution^20^. Vidhyadharan *et al*. reported nanowire-shaped CuO acts as anodic materials for supercapacitive applications with 620 F/g in aqueous electrolyte^19^. However, only few reports are available based on Cu_2_O which acts as supercapacitive electrode material^21^. Xue *et al*. synthesized Cu_2_O with a hollow octahedral structure with the capacitive value of 58.0 Fg^−1^ and Cu_2_O with a core-shell morphology as supercapacitive active material with capacitance value of 88.0 Fg^−1^ ^22^. Patake *et al*. reported the thin film fabrication of the Cu_2_O and used as a supercapacitive electrode with the maximum capacitance value of 36.0 Fg^−1^ ^23^. Thus, although the effects of different morphologies of Cu_2_O as an electrode material on its supercapacitive performance have been investigated, the particle size and shape effect on the supercapacitive properties has not yet been reported significantly. However, the morphologies of other metal oxides and the effect of particle size over the supercapacitive behavior of the electrode materials were investigated. Because the particle size and morphology are known to effect the chemical and physical properties of a active electrode materials, the size and morphology of Cu_2_O would be expected to significantly affect its electrochemical properties. Recently, a morphological study of wire shaped and hollow spherical shaped nanoboxes of Cu_2_O, obtained by varying the synthesis method, was reported. Among these methods, the polyol method is the most popular, because of various advantages such as that it inhibits the agglomeration of particles during synthesis and its ability to control the growth limit^24^.

This method enabled the use of an extensive variety of species such as chemical, polymers, an-ions, bio-molecules, and surfactants which control the size and morphology of the Cu_2_O, as a result of certain above mentioned species inhibited the growth rate of the crystal and preferentially adsorbed on the surface of the specific crystals^25,26^. For examples, cuprous oxides spheres synthesized by the nitrate precursor of copper and poly(vinylpyrrolidone) (PVP) in ethylene glycol (EG), where EG acts as the solvent as well as the stabilizer^27,28^. This led us to develop a cost-effective one-step modified polyol-assisted (metal-organic framework) solvothermal approach to fabricate cuprous oxides with a cube-like morphology, where EG plays the role of solvent, reducing agent, and stabilizer. The effect of the solvothermal-reaction time on the morphology of Cu_2_O was systematically observed in order to rationalize the structural configuration of the Cu_2_O. The growth mechanism of the Cu_2_O with respect to time was investigated in detail. The morphological effect over the electrochemical supercapacitive performance of Cu_2_O were also evaluated in detail. The electrochemical supercapacitive performance results displayed that the electrode consisting of cube-like Cu_2_O displayed high value of the specific capacitance of 365 Fg^−1^ at the current load of 0.75 Ag^−1^, and it retains more than 89% of the capacitance even at higher current density.

## Experimental

### Materials

Copper nitrate was acquired from Sigma Aldrich, South Korea. Ethylene glycol (EG, C_2_H_6_O_2_), poly(vinylpyrrolidone) (PVP), and ethanol were purchased from Duksan-Pure-Chem. Co. Ltd., S. Korea. DI water, which was obtained by using a commercial water filter (PURE ROUP 30), was used in all the experiments.

### Experimental details

Cu_2_O cubes were synthesized via a modified polyol method (metal-organic framework). In the typical synthesis process, 0.1 M copper nitrate and 2.0 g of PVP were thoroughly mixed in 100 mL of EG on magnetic stirrer for approximately 30-min to obtained a thick slurry with EG being both the solvent and stabilizer^28^. The above obtained slurry was poured in a small Teflon lined autoclave system and sealed carefully, after which the entire assembly was placed inside an electric oven and maintained at a temperature of 160 °C. After different time intervals, the resulting red colored precipitate was obtained, washed it rapidly with ethanol-water mixture, dried at 80 °C for 12 h, and stored in desiccator. For the purposes of comparison, the same reaction procedure was followed by varying the reaction time as 8, 10, and 12 h. The resulting product collected at different time intervals, i.e., at 8, 10, and 12 h, are abbreviated as Cu_2_O-8h, Cu_2_O-10h, and Cu_2_O-12h, respectively.

## Methods

The morphological evaluation of Cu_2_O-8h, Cu_2_O-10h, and Cu_2_O-12h was conducted by using scanning electron microscopy (SEM, HITACHI-S4800). Diffraction patterns were obtained from X-ray diffractometer using PANalytical-X-Pert, PRO-MPD-Netherlands system. The chemical behavior and composition, and binding behavior of the cube-like Cu_2_O were obtained over X-ray photoelectron spectroscopy (XPS) using system of the ESCALAB-250. The half cell assembly electrochemical performance of the electrode materials such as CV (cyclic voltammetry) and GCD (galvanostatic charge-discharge) were measured over a Versa STATE 3 workstation. half-cell-electrochemical characteristics, i.e., cyclic voltammetry (CV) and galvanic charge-discharge (GCD), were measured using a Versa STAT 3 USA electrochemical workstation.

### Electrode preparation and half-cell assembly electro-measurements

The electrochemical measurmets of the prepared electrode were used to examined the half-cell assembly electrochemical performance of the electrodes in aqueous 2 M KOH electrolyte. A Ag/AgCl (3 M KCl) and platinum were used as a reference and counter electrodes in the setup cell. Three different working electrodes were fabricated from Cu_2_O-8h, Cu_2_O-10h, and Cu_2_O-12h, respectively, by preparing a slurry using 10% poly(tetrafluoroethylene), 10% carbon black, and 80% of Cu_2_O-8h, Cu_2_O-10h, or Cu_2_O-12h, respectively, as the active material. A coating of the obtained slurry was subsequently applied to commercially available Ni foam and dried it further. CV was accomplished at different scan rate over the potential range of 0–0.5 V, whereas the galvanostatic charge discharge measurement were carried out at different current load in the same 0–0.5 V potential window.

## Results and Discussion

### SEM, XRD, XPS and growth mechanism of the Cu_2_O

**Figure 1:**
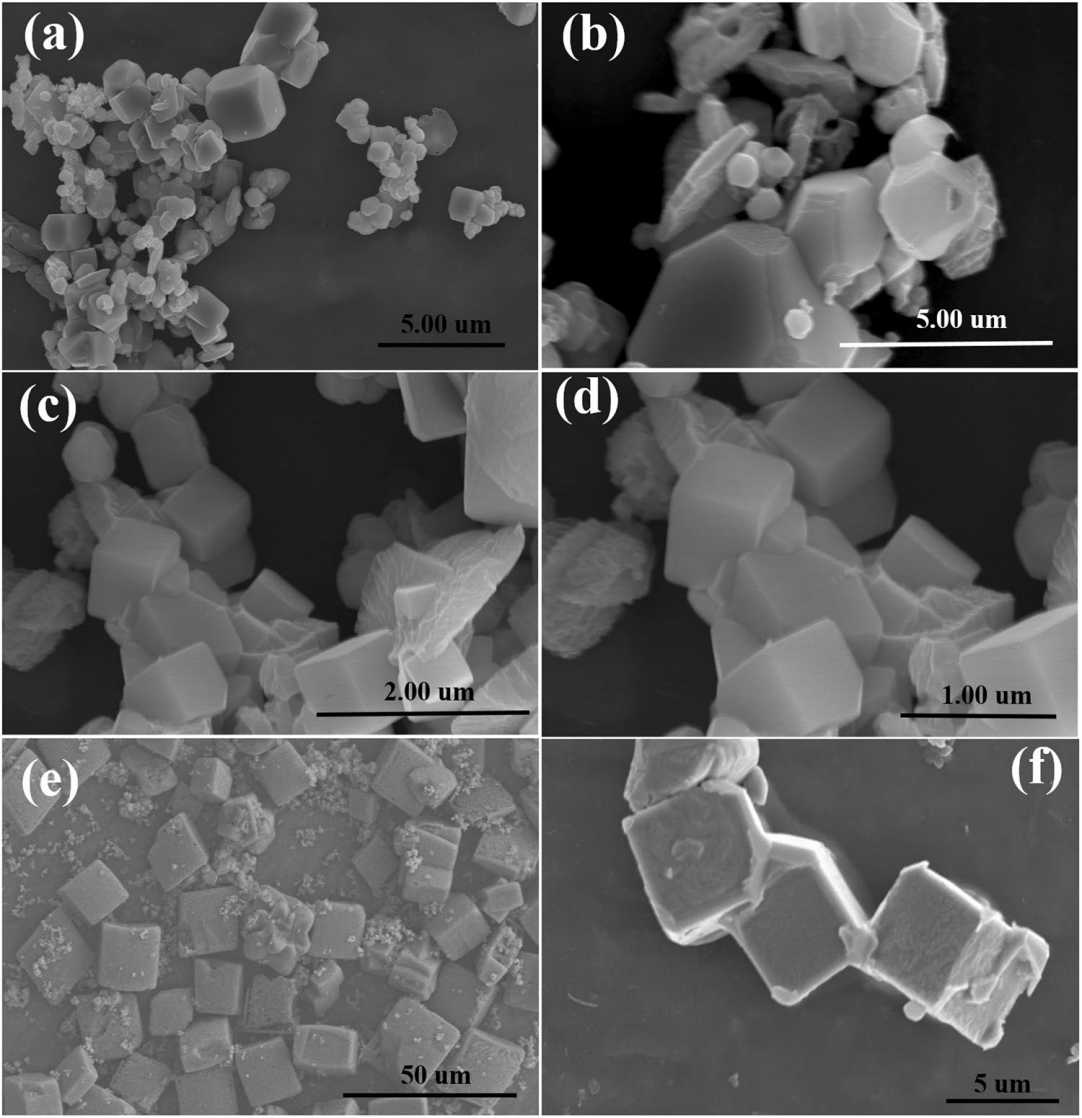
FE-SEM images of the (**a**,**b**) Cu_2_O-8h, (**c**,**d**) Cu_2_O-10h, and (**e**,**f**) Cu_2_O-12h.

For optimization purposes, the morphology of the Cu_2_O was observed after different time intervals, i.e., 8, 10, and 12 h, and the results are presented in Fig. 1. The morphology of the Cu_2_O after the 8-h reaction is not very clear and irregular shapes and sizes can be observed, whereas after increasing the time from 8 h to 10 h, the morphology became more well defined and cubic in shape^28^. However, prolonging the reaction time to 12 h resulted in the cubic-shaped morphology of the Cu_2_O become clearer and more prominent.

**Figure 2:**
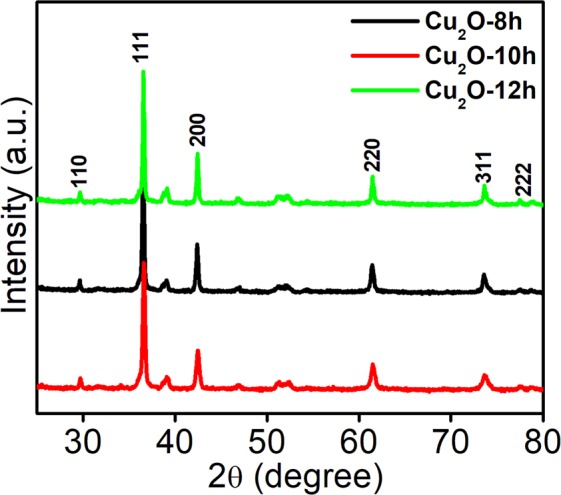
XRD patterns of Cu_2_O-8h, Cu_2_O-10h, and Cu_2_O-12h.

The crystal structure and phase behavior of the Cu_2_O synthesized under different experimental conditions were examined by XRD and results are displayed in Fig. 2. The pattern reveals that the Cu_2_O obtained at 8 h contains some impurities, which might be due to the presence of unreacted compounds in the system because of the short reaction time, which is obvious and confirmed by SEM. The figure shows the diffraction pattern of the optimized Cu_2_O, which exhibits the complete and clear pattern corresponds to the (110), (111), (200), (220), and (311) planes of cuprous oxide, respectively^28–30^. All the diffraction planes are indexed to the face centered cubic cuprous oxide; JCPDS No: 05-0667^30^. The additional peak that appears on the diffraction pattern of the Cu_2_O-8h is due to the short reaction time. However, a few other diffraction peaks are also observed at 12 h of the reaction, and these are indexed to the planes of CuO; hence, it is obvious that the temperature affected the oxidation state of the copper inside the reaction system. These results are in accordance with those of other previously reported work.

**Figure 3:**
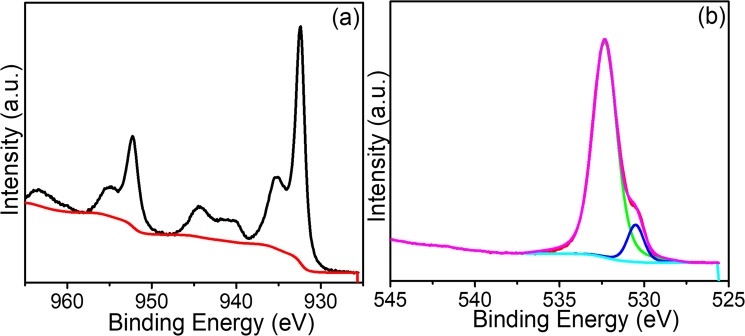
(**a**) High resolution Cu 3p XP spectra and (**b**) O 1 s XP spectra of Cu_2_O-12h.

The electronic state and chemical composition of the copper was investigated by using XPS technique and obtained parameter are presented in Fig. 3 and Fig. S1. The overall survey spectra of the selected Cu_2_O show the presence of copper and oxygen. The visible and intense peak located at the binding energy (BE) of ~932.68 eV and ~952.50 eV (Fig. 3a) corresponds to the Cu 2p_3/2_ and Cu 3p_1/2_, which are also the characteristics peaks of the Cu_2_O particles^29^. The O 1 s graph (Fig. 3b) shows two fitted symmetric peaks located at BE of ~530.65 eV and ~531.98 eV, which corresponds to the presence of the lattice oxygen in Cu_2_O and adsorbed oxygen, respectively^30^.

Generally, the synthesis of Cu_2_O with different morphologies proceeds via either chemical or electrochemical routes to obtain equally sized particles, whereas the exact mechanism is not well established. However, these processes are not necessarily useful to obtain the exact and uniform morphology because of the various steps involved, which might complicate the control of the morphology and uniformity of the particle sizes. To avoid this problem, a polyol-modified method was employed to synthesize cube-like Cu_2_O by using a simple metal-organic framework. It is widely recognized that copper nitrate is easily reduced by EG and that the development of a cuprous phase with various morphologies depends on the template and reaction parameters. The reduction of the Cu follows these two steps^31^:

1 $$2{{\rm{HOCH}}}_{{\rm{2}}}{{\rm{CH}}}_{{\rm{2}}}{\rm{OH}}\to {{\rm{2CH}}}_{{\rm{3}}}{\rm{CHO}}+{{\rm{2H}}}_{{\rm{2}}}{\rm{O}}$$

2 $${{\rm{CH}}}_{{\rm{3}}}{\rm{CHO}}+{\rm{2Cu}}{({{\rm{NO}}}_{{\rm{3}}})}_{{\rm{2}}}+{{\rm{2H}}}_{{\rm{2}}}{\rm{O}}\to {{\rm{CH}}}_{{\rm{3}}}{\rm{COOH}}+{{\rm{4HNO}}}_{{\rm{3}}}+{{\rm{Cu}}}_{{\rm{2}}}{\rm{O}}$$

**Figure 4:**
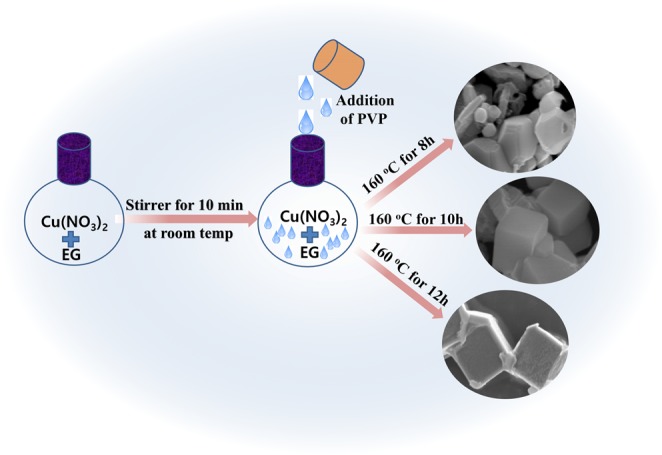
Schematic diagram of the synthesis of the cubic Cu_2_O.

In the initial reaction, the copper ions are reduced by the decomposed EG at higher temperature, as shown in the above reaction steps, and crystalline Cu_2_O is rapidly formed (Fig. 4). The surfaces of these new Cu_2_O crystals have highly indexed crystallographic planes, which are responsible for causing particle aggregation and reduces the energy of the surface of the planes.

The polyhedral particles grown along the different direction at different growth rates in the beginning of the reaction, which is due to the surface energy differences.

Previous reports clearly mention that the ratio of the growth rate, i.e., R of the planes (110 and 111) direction, take the geometry of the cuprous oxide crystal into consideration. The symmetry of the Cu_2_O will be cubic when the value of R = 0.58. Therefore, in the present case, the development of cube-like Cu_2_O initiated by the different rate of adsorption of nitrate and PVP on the plane surface (111 and 111), determine the rates of the different growth of these two faces of the crystal. Moreover, the copper anion plays a vital role to determine the morphology of the Cu_2_O. In present case, EG acts as the solvent and as well as the reducing agent, whereas PVP is used as capping agent and facilitates the formation of the uniform cube-like structure of the Cu_2_O^32^. In other words, the dispersing medium and reducing nature provided by the EG and the colloidal stabilization facilitated by the PVP are helpful to initiate the color change during reaction, which marks the different stages and indicate the reduction of the copper ions.

### Electrochemical supercapacitive performance of the fabricated electrodes

**Figure 5:**
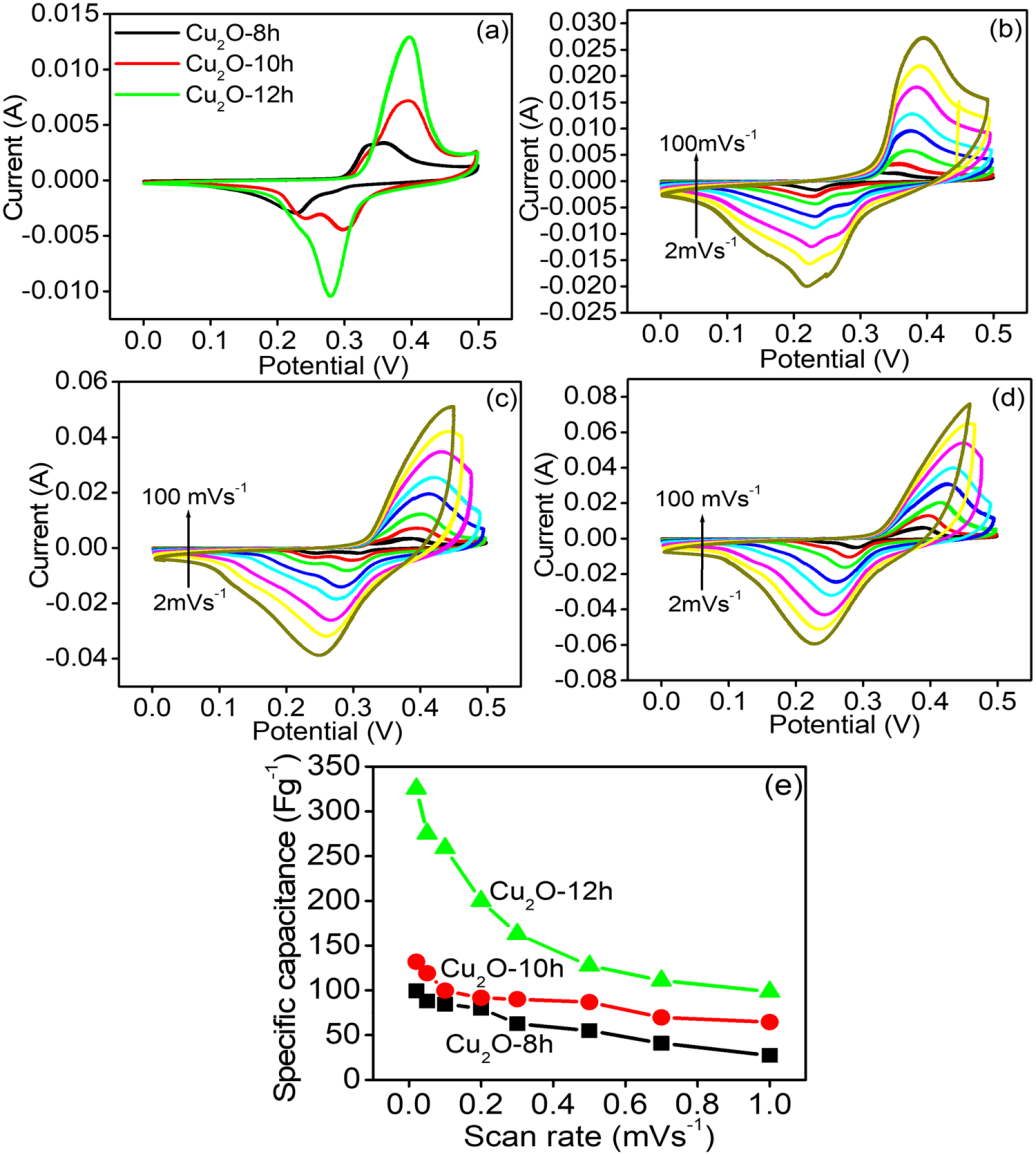
(**a**) Comparative CV curve of Cu_2_O-8h, Cu_2_O-10h and cube like Cu_2_O-12h at 2 mVs^−1^, (**b**) CV curve of Cu_2_O-8h, (**c**) CV curve of Cu_2_O-10h, (**d**) Cu_2_O-12h at different scan rate (**e**), and specific capacitance at various scan rate.

We examined the morphological effect of Cu_2_O on its performance as an electrochemical energy storage device by performing CV and charge/discharge analysis, which is a very important tool to inspect the capacitance of an electrode material. First, we performed the CV analysis, which is a necessary and very common prominent tool to examine the oxidation-reduction behavior and govern the capacitance capability of the developed electrode. All the electrochemical experiments were performed by using a three-assembly cell system in 2 M KOH electrolyte. Figure 5a represents the comparative cyclic voltammetry curves of Cu_2_O-8h, Cu_2_O-10h, and cube-like Cu_2_O-12h in the 0.0–0.5 V potential range and the scan rate of 5 mV/s. The CV curves of Cu_2_O-8h, Cu_2_O-10h, and cube-like Cu_2_O-12h are shown in Fig. 5b–d at the various scan rate ranging from 5 to 100 mV/s. Each of the CV profiles clearly show the anodic and cathodic peaks, indicating that the Cu_2_O redox reaction occurred during the electrochemical energy storage procedure. These redox peaks also indicate that the electrochemical energy storage mechanism is supported by the pseudocapacitance behavior. This behavior is completely different from that of EDL capacitors, of which the CV curves have an almost rectangular shape.

As shown in Fig. 5a–d the pseudocapacitive nature of Cu_2_O electrode is due to the transition of the oxidation sate of the copper from Cu (I) to Cu (II) and vice versa. Furthermore, as demonstrated by the cyclic voltammetry profile the anodic peak seemed to be the result of the oxidation of the Cu_2_O electrodes to Cu(OH)_2_ and CuO^21^. The presence of the cathodic peaks are owing to the transformation of the CuO and Cu(OH)_2_ in Cu_2_O through the reduction process. The reaction between the Cu(I) and Cu(II) are attributed to the following equation:

3 $${{\rm{Cu}}}_{{\rm{2}}}{\rm{O}}+{{\rm{2OH}}}^{-}\leftrightarrow {\rm{2CuO}}+{{\rm{H}}}_{{\rm{2}}}{\rm{O}}+{{\rm{2e}}}^{-}$$

4 $${\rm{Cu}}{({\rm{OH}})}_{{\rm{2}}}+{{\rm{2e}}}^{-}\leftrightarrow {{\rm{2Cu}}}_{{\rm{2}}}{\rm{O}}+{{\rm{2OH}}}^{-}+{{\rm{H}}}_{{\rm{2}}}{\rm{O}}$$

In a pseudocapacitive material, the relation between the anodic and cathodic current and the scan rate is highly important to determine whether the surface specific-capacitance arises from the bulk diffusion or oxidation-reduction reaction. In the present case, Fig. 5a–d show an almost linear relationship which suggest that the diffusion rate was controlled during the electrochemical process.

Figure 5a clearly shows that the CV curve of cube-like Cu_2_O-12h occupies an area larger than that of Cu_2_O-8h and Cu_2_O-10h under the same scan rate window, indicating the better electrochemical capacitance performance. Furthermore, the CV curves of Cu_2_O-8h, Cu_2_O-10h, and cube-like Cu_2_O-12h at different scan rates are depicted in Fig. 5a–d. In each case, the CV curve shows an electrochemical response that increases linearly with higher scan rates. After the scan rate was increased, the oxidation peaks was shifted to the positive potential and the reduction peaks shifted to the negative potential. These peak shifts are due to the internal resistance over the electrode and confirm the pseudocapacitive behavior of the Cu_2_O microstructures^33^. These shifts also confirmed the charge diffusion polarization in the pseudocapacitive lecetrode^34^. The fact that the current response increases with the scan rates suggest that the kinetics of the interfacial faradic oxidation/reduction reaction and the rate of the ionic and electronic response are expressively faster even at high scan rates^35^. Figure 5e indicates that the specific-capacitance of Cu_2_O microstructures gradually decreases as the scan rate increases. The lower specific-capacitance values at higher current scan rate is attributed to the lower degree of diffusion of the electrolytic ionic solution over the surface of the electrodes. However, at lower current scan rate, diffusion of the electrolytes increases, resulting in higher specific capacitance. Analysis of the CV curve reveals that two cathodic peaks appeared for the Cu_2_O microstructures, which indicates that multistep intercalation of Cu^+^ at additional electrochemical sites occurs.

The specific capacitance of Cu_2_O electrodes was derived from the CV curves and using following equations:

5 $${{\rm{C}}}_{{\rm{sp}}}=\int \mathrm{IdV}/({\rm{\Delta }}\mathrm{VM}{\rm{\nu }})$$

where C_sp_ is the value of the specific capacitance of the evaluated sample, V is the potential, I is average current during the anodic and cathodic sweep, and M is amount of the active material loading over the surface of the electrode.

I is the average current during the cathodic and anodic sweep, V is the potential, and M is the weight of the active material. The values of the specific capacitance of Cu_2_O-8h, Cu_2_O-10h, and cube-like Cu_2_O-12h calculated from the CV curve analysis are 101.4 Fg^−1^, 142 Fg^−1^, and 325.5 Fg^−1^, respectively at a scan rate of 5 mV/s. Cube-like Cu_2_O-12h exhibited higher specific capacitance.

**Figure 6:**
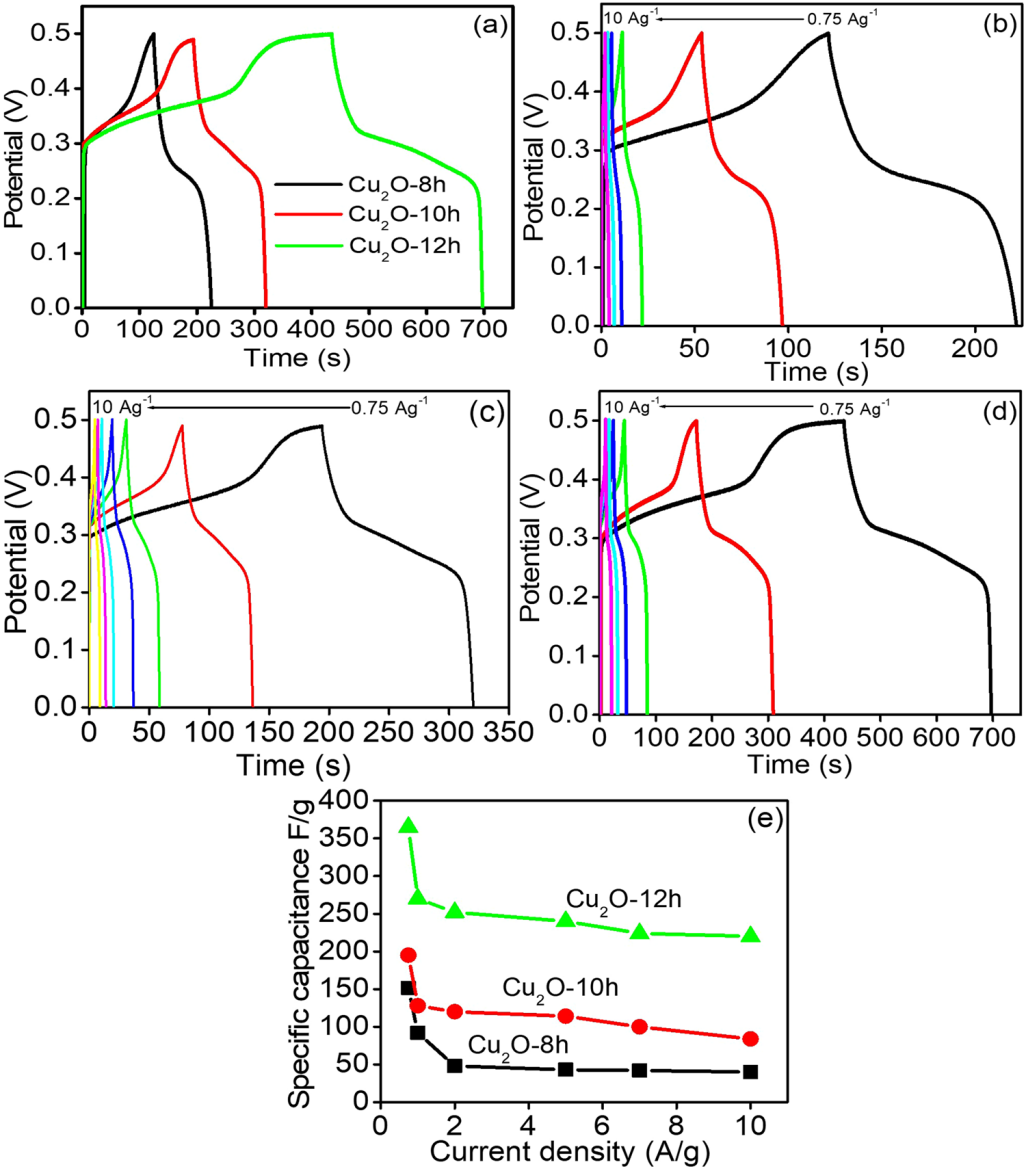
(**a**) Comparative CD curve of Cu_2_O-8h, Cu_2_O-10h and cube like Cu_2_O-12h at 0.75 Ag^−1^, (**b**) CD curve of Cu_2_O-8h, (**c**) CD curve of Cu_2_O-10h, (**d**) CD curve of Cu_2_O-12h at different current density in 2 M KOH and (**e**) calculated specific capacitance at various current density.

The GCDs of the Cu_2_O-8h, Cu_2_O-10h, and cube-like Cu_2_O-12h at a fixed current load of 0.75 Ag^−1^ and different current loads of 1, 2, 5, 7, and 10 Ag^−1^ are represented in the Fig. 6a–d. The cube-like Cu_2_O-12h exhibited much higher capacitive performance of ~365 Fg^−1^ at the current load of 0.75 Ag^−1^ than Cu_2_O-8h and Cu_2_O-10h (~151 and ~195 Fg^−1^) and this might be due to its unique cube-like morphology. In addition, the GCDs of Cu_2_O-8h, Cu_2_O-10h, and cube-like Cu_2_O-12h at different current loads are presented in Fig. 6b–d. The specific capacitive value of the Cu_2_O microstructures were obtained using following equation:

6 $${{\rm{C}}}_{{\rm{sp}}}={\rm{I}}\,\mathrm{dt}/\mathrm{mdv}$$

where C_sp_ is the value of the specific-capacitance in Fg^−1^, dt is the discharging time, dv is the upper and lower potential differences, and m is the loading amount of the active material over the electrode. The specific capacitance of cube-like Cu_2_O-12h (Fig. 6d) was ~365, ~270, ~252, ~240, ~224, and ~220 Fg-1 at the current loads of 0.75, 1, 2, 5, 7, and 10 Ag^−1^ respectively. This result shows that higher storage energy capacity occurs at low current because of improved intercalation of the electrolytic solution during the GCDs experiment. In comparison, the specific capacitance for Cu_2_O-10h was ~195, ~128, ~120, ~114, ~100, and ~84 Fg^−1^, respectively (Fig. 6c). Similarly, the specific capacitance of Cu_2_O-8h was ~151, ~92, ~58, ~47, ~40, and ~35 Fg^−1^ at current density of 0.75, 1, 2, 5, 7, and 10 Ag^−1^ respectively (Fig. 6b). In particular, cube-like Cu_2_O-12h exhibited excellent capacitive performance and rate capability as compared to Cu_2_O-8h and Cu_2_O-10h due to its three dimensional architecture, which provides number of electrochemically active sites for intercalation of the maximum number of the electrolyte ions during the reaction and can maximize its exploitation as an effective electrode materials.

Moreover, as presented in Fig. 6e the optimal specific capacitive value of the cube-like Cu_2_O-12h retained 220 Fg^−1^ even at higher current load of 10 Ag^−1^, where as much as 60% of the capacitance was retained as compared to that measured at 0.75 Fg^−1^.

In contrast, Cu_2_O-10h and Cu_2_O-8h retained only 45% and 23% of its initial specific capacitance when the current load was increased by the same amount. This clearly indicates that the capacitance retention of Cu_2_O-10h and Cu_2_O-8h is less than 50%, demonstrating that the cube-like Cu_2_O-12h electrode is much more stable even at higher current density. The decreasing the value of the specific capacitance with increasing current loads obviously occurred because, at higher current density, the electrode has a short time in which to interact with the electrode. As shown in the figure, an apparent plateau region is observed at highr voltage of 0.31 V at low current load of 1 Ag^−1^, which might be due to the oxidation of Cu_2_O to Cu(OH)_2_, and which also corresponds to the CV results.

Table 1 presents a comparison of cube-like Cu_2_O-12h with previously reported Cu_2_O based electrodes material. As compared to the previous works, the cube like Cu_2_O-12h outperformed most of the previous capacitors.

**Table 1:** Comparison of the capacitance of cube-like Cu_2_O-12h with that of other reported Cu_2_O-based morphological structures.

S.No	Structural morphology	Electrolyte	C_sp_ (Fg^−1^)	Current density A g^−1^	Cyclic stability		ref.
					Cycle Number	Retention%	
1	RGO/Cu_2_O	1 M KOH	98.5	1	1000	50%	^7^
2	Cu_2_O/CuO/Co_3_O_4_	3 M KOH	318	0.5	3000	80%	^8^
3	Cu_2_O microsphere	2 M KOH	144	0.1	100	99%	^36^
4	cubic Cu_2_O	2 M KOH	132	0.1	100	99%	^36^
5	CuO NR_s_	1 M Na_2_SO_4_	206.6	1	1000	88%	^37^
6	rGO/Cu_2_O	1 M Na_2_SO_4_	195	2	1000	92%	^38^
7	CuO-rGO	2 M KOH	296	1	2000	96.1%	^39^
8	Cu_2_O/CuO/RGO	6 M KOH	173.4	1	100,000	98.2	^40^
9	Cu/Cu_2_O nanoparticles	2 M KOH	380	1 mA/cm^2^	5000	96%	^41^
10	Cube-like Cu_2_O	2 M KOH	402	0.75	2500	89.5%	Present case

**Figure 7:**
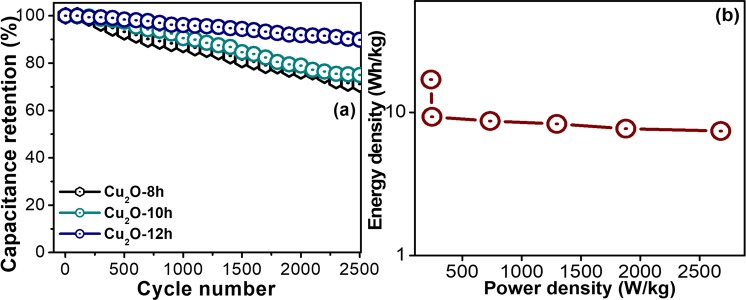
(**a**) Capacitance retention V_s_ number of charge-discharge cycles of the Cu_2_O-8h, Cu_2_O-10h, and Cu_2_O-12h, and (**b**) Ragone plot of the Cu_2_O-12h.

Apart from the specific capacitance and other behaviors, the cyclic performance of electrode materials are also an important key parameter that relates to the supercapacitive performance. The cube-like Cu_2_O-12h electrode was used for the consecutive charge/discharge cyclic stability test during which the current rate was kept constant. Figure 7a displays the retention of the capacitive value for 2500 cycles as a function of the number of cyclic runs. The figure shows that the capacitive retention is dramatically decreased in the first few hundred cycles, which might be attributed to the saturation of active sites on the surface of the Cu_2_O electrodes during consecutive charge/discharge cycling. Furthermore, an increase and subsequent gradual decrease in the capacitance retention is detected until 2500 cycles. This slight decrease in capacitance is mainly due to structural breakdown and delamination of Cu_2_O on the nickel foam surface in aqueous solution. The electrolytic ions may be trapped in the Cu_2_O because of repeated charging/discharging. Interestingly, excellent cycling stability was maintained with the capacity retention approximating 89.5% even after 2500 cycles which can provide greater cycling stability compared to the previously reported Cu_2_O-based electrode materials prepared by a different synthesis method. This indicates its suitability for hydrothermally synthesized Cu_2_O electrode material. The cube-like Cu_2_O-12h electrode highlights the excellent cycling stability of this material and indicates the better reversibility and excellent electrochemical stability due to its unique cubic morphology. In addition, it also exhibited significantly longer discharging and charging time, suggesting that a large number of electrolytes ions and electrons contributed to the charging and discharging processes.

The power and energy density performance of the cube-like Cu2O-12h was analyzed by obtaining the Ragone plot or by calculating the energy density versus the energy and power density using the following equations;

7 $${\rm{E}}={1/\mathrm{2CV}}^{{\rm{2}}}$$

8 $${\rm{P}}=E/t$$

Figure 7b clearly demonstrates that the energy density decreases when the power density increases, which is similar to the result that obtained from the specific capacitance section. However, the power density increases as the current density increases. This is because of the ions of the electrolytes have the limited and short period of time for the accessibility of the pores of surface of the electrodes during the fast charging at higher current loads, whereas, at lower current density, pores can be fully utilized by the ions due to the lower ionic diffusion process. The Cu_2_O-12h electrode exhibits an energy density of 16.95 Wh/Kg at a power density of 235.4 W/Kg and higher power density of 2678.5 W/Kg at a lower energy density of 7.5 Wh/Kg. These higher power and energy densities are direct consequences of the high rate capability of the Cu2O-12h electrode.

Overall, the enhanced electrochemical supercapacitive performances, such as the high specific capacitance and robust life span of the cube-like Cu_2_O is attributed to the following: the main important factor is the morphology of the cube-like Cu_2_O, which shorten the diffusion rate of electrolytic ions and electrons, thereby reducing the internal and charge transfer resistance. Second, the higher conductive behavior of the nickel foam with unique three- dimensional architect easily provides a faster electron transfer through various channels and also provides various active site for the electrochemical reactions. This also enables the ions of the electrolytes to participate in the fast oxidation-reduction reaction and displays strong bond with the current collector to improve the overall electrochemical performance. Furthermore, crystallinity plays an important factor in supercapacitive applications. The Cu_2_O microstructures studied in the present work have a single crystalline nature with fewer grain boundaries and this helps to protect their morphology during electrochemical reactions.

## Conclusions

In summary, a one-step and simple modified polyol-assisted method was used for the fabrication of cubic Cu_2_O in ethylene glycol as solvent, which also acted as reducing and stabilizing agent. The effect of the reaction and its associated behavior were studied systematically by using standard characterization techniques. The performance of Cu_2_O as an electrochemical supercapacitor was examined in a three-electrode assembly cell. The Cu_2_O-12h (C_sp_ ~365 Fg^−1^) exhibited enhanced capacitive performance and cyclic stability compared to Cu_2_O-8h (C_sp_ ~151 Fg^−1^) and Cu_2_O-10 (C_sp_ ~195 Fg^−1^). These properties are essential for employing electrode materials in supercapacitive applications. The cubic-like morphology, which shorten the diffusion times of the electrolytic ions and electrons, reduces both the internal and charge transfer resistance during the electrochemical process. The excellent electrochemical supercapacitive performance of the electrode, which was fabricated by a facile and scalable synthesis method, presents a worthy choice for electrodes used in energy storage applications.

## Supplementary information

Supporting Information

## References

[1] Wang G, Zhang L, Zhang J (2012). A review of electrode materials for electrochemical supercapacitors. J. Chem. Soc. Rev..

[2] Kim JH, Zhu K, Yan Y, Perkins CL, Frank AJ (2010). Microstructure and pseudocapacitive properties of electrodes constructed of oriented NiO-TiO_2_ nanotube arrays. Nano Lett.

[3] Wang HL, Casalongue HS, Liang YY, Dai HJ (2010). Ni(OH)_2_ Nanoplates grown on graphene as advanced electrochemical pseudocapacitor materials. J. Am. Chem. Soc..

[4] Zhao G (2015). Synthesis and lithium-storage properties of MnO/reduced graphene oxide composites derived from graphene oxide plus the transformation of Mn(VI) to Mn(II) by the reducing power of graphene oxide. J. Mater. Chem. A.

[5] Barai HR, Rahman MM, Joo SW (2017). Template-free synthesis of two-dimensional titania/titanate nanosheets as electrodes for high-performance supercapacitor applications. Journal of Power Sources.

[6] Barai HR, Rahman MM, Joo SW (2017). Annealing-free synthesis of K-doped mixed-phase TiO_2_ nanofibers on Ti Foil for electrochemical supercapacitor. Electrochim. Acta.

[7] Kuang M (2015). Hierarchical Cu O/CuO/Co_3_O_4_ core-shell nanowires: synthesis and electrochemical properties. Nanotechnology.

[8] Dong X (2014). Direct synthesis of RGO/Cu_2_O composite films on Cu foil for supercapacitors. J. Alloys and Compounds..

[9] Zhao G (2014). Two-dimensional Cr_2_O_3_ and interconnected graphene-Cr_2_O_3_ nanosheets: synthesis and their application in lithium storage. J. Mater. Chem. A.

[10] Parveen N, Ansari SA, Cho MH (2017). Intercalated reduced graphene oxide and its content effect on the supercapacitance performance of the three dimensional flower-like β-Ni(OH)_2_ architecture. New Journal of Chemistry.

[11] Parveen N (2018). Facile synthesis of SnS_2_ nanostructures with different morphologies for high-performance supercapacitor applications. ACS Omega.

[12] Parveen N (2018). Solid-state symmetrical supercapacitor based on hierarchical flower-like nickel sulfide with shape-controlled morphological evolution. Electrochimica Acta.

[13] Zang Z, Nakamura A, Temmyo J (2013). Nitrogen doping in cuprous oxide films synthesized by radical oxidation at low temperature. Mater. Lett..

[14] Ke FS (2009). One-step fabrication of CuO nanoribbons array electrode and its excellent lithium storage performance. Electrochim. Acta.

[15] Dubal DP, Gund GS, Holze R, Lokhande CD (2013). Mild chemical strategy to grow micro-roses and micro-woolen like arranged CuO nanosheets for high performance supercapacitors. J. Power Sources.

[16] Chen K, Song S, Xue D (2015). Faceted Cu_2_O structures with enhanced Li-ion battery anode performances. CrystEngComm.

[17] Heng B (2013). Rapid synthesis of CuO nanoribbons and nanoflowers from the same reaction system, and a comparison of their supercapacitor performance. RSC Adv..

[18] Zhang YX, Huang M, Li F, Wen ZQ (2013). Controlled synthesis of hierarchical CuO Nanostructures for electrochemical supercapacitor. Inter. J. Electrochem. Sci.

[19] Vidyadharan B, Misnon I, Ismail J, Yusoff MM, Jose R (2015). High performance asymmetric supercapacitors using electrospun copper oxide nanowires anode. J. Alloys Compd..

[20] Navathe GJ (2015). Rapid synthesis of nanostructured copper oxide for electrochemical supercapacitor based on novel [HPMIM][Cl] ionic liquid. J. Electroanal. Chem..

[21] Dong Y (2014). 3D binder-free Cu_2_O@Cu nanoneedle arrays for high-performance asymmetric supercapacitors. J. Mater. Chem. A.

[22] Chen KF, Song SY, Xue DF (2013). Chemical reaction controlled synthesis of Cu_2_O hollow octahedra and core–shell structures. CrystEngComm.

[23] Patake VD, Joshi SS, Lokhande CD, Joo Q-S (2009). Electrodeposited porous and amorphous copper oxide film for application in supercapacitor. Mater. Chem. Phys..

[24] Zhu PL, Zhang JW, Wu ZS, Zhang ZJ (2008). Microwave-assisted synthesis of various ZnO hierarchical nanostructures: effects of heating parameters of microwave oven. Cryst. Growth Des..

[25] Ho JY, Huang MH (2009). Synthesis of submicrometer-sized Cu2O crystals with morphological evolution from cubes to hexapod structures and their comparative photocatalytic activity. J. Phys. Chem. C.

[26] Kowalczyk B (2012). Charged nanoparticles as supramolecular surfactants for controlling the growth and stability of microcrystals. Nat. Mater..

[27] Hong X (2009). Synthesis of sub-10 nm Cu2O nanowires by poly (vinyl pyrrolidone)-assisted electrodeposition. J. Phys. Chem. C.

[28] James S (2018). Nanostructured cuprous-oxide-based screen-printed electrode for electrochemical sensing of picric acid. Journal of Electronic Materials.

[29] Zhou L (2013). Facile synthesis of highly stable and porous Cu_2_O/CuO cubes with enhanced gas sensing properties. Sensors and Actuators B.

[30] Ji R, Sun W, Chu Y (2014). One-step hydrothermal synthesis of Ag/Cu_2_O heterogeneous nanostructures over Cu foil and their SERS applications. RSC Adv..

[31] Kim MH, Lim B, Lee EP, Xia Y (2008). Polyol synthesis of Cu_2_O nanoparticles: use of chloride to promote the formation of a cubic morphology. J. Mater. Chem..

[32] Li Q (2013). Structure-dependent electrocatalytic properties of Cu_2_O nanocrystals for oxygen reduction reaction. J. Phys. Chem. C.

[33] Cai D (2013). Comparison of the Electrochemical Performance of NiMoO_4_ nanorods and hierarchical nanospheres for supercapacitor applications. ACS Appl. Mater. Interfaces.

[34] Liu J (2014). A flexible alkaline rechargeable Ni/Fe battery based on graphene foam/carbon nanotubes hybrid film. Nano Lett..

[35] Zhang F (2013). Flexible films derived from electrospun carbon nanofibers incorporated with Co_3_O_4_ hollow nanoparticles as self‐supported electrodes for electrochemical capacitors. Adv. Funct. Mater..

[36] Chen L (2015). Copper salts mediated morphological transformation of Cu_2_O from cubes to hierarchical flower-like or microspheres and their supercapacitors performances. Scientific Reports.

[37] Nathan DM (2018). One-pot hydrothermal preparation of Cu_2_O-CuO/rGO nanocomposites with enhanced electrochemical performance for supercapacitor applications. Applied Surface Science.

[38] Park H, Han TH (2014). Facile hybridization of graphene oxide and Cu_2_O for high-performance electrochemical supercapacitors. Macromol. Res..

[39] Liu A (2016). Hierarchical dandelion-like copper oxide wrapped by reduced graphene oxide: Hydrothermal synthesis and their application in supercapacitors. Mater. Res. Bull..

[40] Wang K, Dong X, Zhao C, Qian X, Xu Y (2015). Facile synthesis of Cu_2_O/CuO/RGO nanocomposite and its superior cyclability in supercapacitor. Electrochimica Acta.

[41] Prasad KPS (2013). Post-synthetic functionalization of mesoporous carbon electrodes with copper oxide nanoparticles for supercapacitor application. Microporous and Mesoporous Materials.

