# Supplementary material for: Self-assembled Cube-like Copper Oxide Derived from a Metal-Organic Framework as a High-Performance Electrochemical Supercapacitive Electrode Material
Source: Sci Rep. 2019 Jun 24;9:9140. doi: 10.1038/s41598-019-45557-6 (PMC6591407; doi:10.1038/s41598-019-45557-6)
Supplement: Supplementary file 1 — Supporting Information [file 41598_2019_45557_MOESM1_ESM.pdf]

Supporting Information

Self-assembled Cube-like Copper Oxide Derived from a Metal-Organic Framework as a High-Performance Electrochemical Supercapacitive Electrode Material

Abdullah Aljaafari[1], Nazish Parveen[2], Faheem Ahmad[1], Mir Waqas Alam[1], and Sajid Ali Ansari[1*]

[1]Department of Physics, College of Science, King Faisal University, Al-Ahsa 31982, Kingdom of Saudi Arabia

[*]Corresponding author: E-mail address: sansari@kfu.edu.sa

[2]Department of Chemistry, College of Science, King Faisal University, Al-Ahsa 31982, Kingdom of Saudi Arabia

XPS survey spectra of Cu_2O

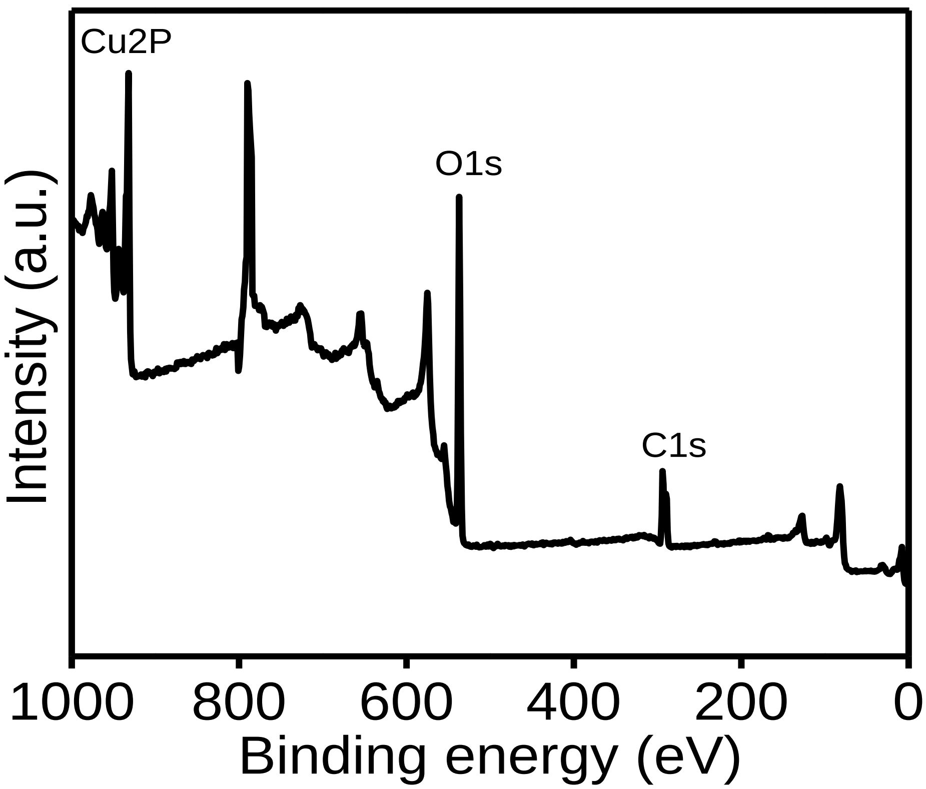

Figure S1. XPS survey spectra of Cu_2O.
